# A general method for combining different family-based rare-variant tests of association to improve power and robustness of a wide range of genetic architectures

**DOI:** 10.1186/s12919-016-0024-y

**Published:** 2016-10-18

**Authors:** Alden Green, Kaitlyn Cook, Kelsey Grinde, Alessandra Valcarcel, Nathan Tintle

**Affiliations:** 1 Department of Statistics, Harvard University, Cambridge, MA 02138 USA; 2 Department of Mathematics and Statistics, Carleton College, Northfield, MN 55057 USA; 3 Department of Biostatistics, University of Washington, Seattle, WA 98195 USA; 4 Department of Statistics, University of Connecticut, Storrs, CT 06269 USA; 5 Department of Mathematics, Statistics, and Computer Science, Dordt College, Sioux Center, IA 51250 USA

## Abstract

Current rare-variant, gene-based tests of association often suffer from a lack of statistical power to detect genotype–phenotype associations as a result of a lack of prior knowledge of genetic disease models combined with limited observations of extremely rare causal variants in population-based samples. The use of pedigree data, in which rare variants are often more highly concentrated than in population-based data, has been proposed as 1 possible method for enhancing power. Methods for combining multiple gene-based tests of association into a single summary *p* value are a robust approach to different genetic architectures when little a priori knowledge is available about the underlying genetic disease model. To date, however, little consideration has been given to combining gene-based tests of association for the analysis of pedigree data. We propose a flexible framework for combining any number of family-based rare-variant tests of association into a single summary statistic and for assessing the significance of that statistic. We show that this approach maintains type I error and improves the robustness, to different genetic architectures, of the statistical power of family- and gene-based rare-variant tests through application to simulated phenotype data from Genetic Analysis Workshop 19.

## Background

Over the past decade, the rapid decrease in costs for DNA sequence data have made it possible to consider the association of rare single nucleotide variants (SNVs) and complex disease phenotypes. However, the power of any single variant test of genetic association on such rare variants is limited. As such, many gene-based tests of association (simultaneously testing all variants within a gene) have recently been proposed, with the intent to improve statistical power over single marker association tests. Recent insights have classified most of these gene-based tests of association into 2 broad classes: burden (alternatively: collapsing, length, linear) tests and variance component (alternatively: joint, quadratic) tests [1]. Burden tests collapse rare-variant signal into a single measure of total rare-variant “burden,” which is then tested for association with the phenotype. Variance component tests determine the strength of association of a particular variant site with observed trait values, and then aggregate these associations across all variants in the gene.

Despite improvements over single-marker approaches, gene-based tests may still have limited utility in detecting causal rare variants because of an overall lack of power. This lack of power is exacerbated by the fact that optimizing the limited power of gene-based tests of association (by selecting the appropriate burden or variance component test) requires some a priori knowledge of the underlying genetic architecture. In particular, burden tests are most powerful when the variants within a SNV set are mostly causal and have the same causal direction, whereas variance component tests perform optimally in circumstances where the causal direction varies (ie, in the presence of both risk-reducing and risk-increasing SNVs) [1], even for family data [2].

In response, new tests of association have been proposed that simultaneously leverage the strengths of both burden and variance component tests [3–5]. These methods generally propose computing a burden test p-value and a variance components test p-value on the same SNV set, then using some method to combine the individual *p* values from the gene-based tests of association into one summary *p* value for the gene, but are frequently limited by not being applicable to family-based data. One exception is the seqMeta package in *R* [6].

The use of family/pedigree data has become popular as another means of increasing statistical power to detect causal rare variants. Rare SNVs are more concentrated in affected families, meaning that causal SNVs may aggregate in pedigree data more than in the general population or in a traditional case-control study. In this article, we propose a general method of combining different rare-variant tests for use on family-based data. We then quantify to what extent combining family-based rare-variant tests of association increases power and maintains type I error rate for simulated phenotypes using data from Genetic Analysis Workshop 19 (GAW19).

## Methods

### General combination strategy

Our approach, which extends the approach by Derkach et al [5] to the case of family-based data, combines *p* values across a variety of family-based gene-level rare-variant tests of association into a single summary statistic. Given a set of *k* distinct family- and gene-based rare-variant tests of association, the method involves the following 5 steps: (a) Generate a vector of test statistics ${\boldsymbol{Q}}^{(0)}$ = (${Q}_1,\dots,\;{Q}_k\Big)$, where ${Q}_i$ represents the test statistic for the *i* ^*th*^ family-based rare-variant test of association (*i* = 1, …, *k*). (b) Permute the phenotype of interest *m* times. Find ${\boldsymbol{Q}}^{(1)}, \dots,\;{\boldsymbol{Q}}^{(m)},$ where ${\boldsymbol{Q}}^{(j)}$ is the vector of test statistics generated from the *j* ^*th*^ permutation (*j* = 1,…, *m*). (See next paragraph for consideration of pedigree structure.) (c) Convert each test statistic vector, ${\boldsymbol{Q}}^{\left(\boldsymbol{j}\right)}$, to a corresponding vector of *p* values, where ${\boldsymbol{p}}^{(j)}=\left({p}_1^{(j)}, \dots,\;{p}_k^{(j)}\right)$. ${p}_i^{(j)}$ is calculated empirically, with ${p}_i^{(j)}=\frac{rank\left({Q}_i^{(j)}\right)}{m}$. We let $rank\left({Q}_i^{(j)}\right)$ = 1 for the most extreme value of ${Q}_1$ across all *m* permutations, and $rank\left({Q}_i^{(j)}\right)=m$ for the smallest value of ${Q}_i$ across all permutations. (d) Find all ${S}^{(j)}=f\left({\boldsymbol{p}}^{(j)}\right)$, where *S* is a univariate statistic calculated by combining ${p}_1^{(j)},\dots, {p}_k^{(j)}$ for the *j* ^*th*^ permutation. In this article we use ${S}^{(j)}=f\left({\boldsymbol{p}}^{(j)}\right)= \arg \min \left\{{p}_1^{(j)},\dots, {p}_k^{(j)}\right\}$, although other *p* value combination options are possible. (e) Compute the significance level of ${S}^{(0)}$ by finding the percentage of ${S}^{(j)}$ that are greater than ${S}^{(0)}$, out of *m*.

Permuting phenotype values when pedigree structure exists in the samples fails to maintain appropriate type I error rates. To address this limitation, we modify the approach listed in the previous paragraph by first fitting a linear mixed effects model to the desired covariates, with familial correlation as a random effect, and the phenotype of interest as the response, as has been done in related settings [7]. We estimated fixed effects parameters for covariates, and the random effects of familial correlation assuming a covariance matrix proportional to the prespecified kinship matrix. Because the mixed effects model generates predicted values that account for familial status, the residuals are independent of kinship, and therefore independent between subjects. These residuals can then be permuted among the individuals, and the test statistics reevaluated with these “new” phenotypes. Thus, in the previous paragraph, for family data, each ${\boldsymbol{Q}}^{(i)}$ is a function of the residuals from random effects models.

### Application

As a proof-of-concept, we applied the above method to data from GAW19. The data consists of real genotype data, on which blood pressure phenotypes were simulated, for 849 Mexican American individuals across 20 separate pedigrees. We considered the continuous response variable mean arterial pressure (MAP), calculated as (2/3)*(diastolic blood pressure) + (1/3)*(systolic blood pressure). We assessed the relationship between MAP and the 30 genes on chromosome 3 that possess at least 1 casual variant using 1000 permutations per test. Power was then calculated as the proportion of times among the 200 individual phenotype simulations where each gene was found to be significant, using a 0.05 threshold. Type I error was assessed in a similar method on the same 30 genes, but with the trait Q1 (which was simulated to be heritable although unrelated to genotype) as the phenotype of interest.

### Tests used

We examined 8 different family-based rare variant tests of association, and compared their individual performance to the performance of the 8 tests combined. The 8 tests included 4 burden tests and 4 variance component tests, each of which varied in 2 respects: the choice of variant weighting system, and the power to which the score statistic for the *i* ^*th*^ variant, ${U}_i$, was raised. In this case, ${U}_i={\left(y-X\widehat{B}\right)}^{\prime }{\widehat{\Sigma}}^{-1}{g}_i$, where $y$ is the phenotype vector, $X$ the covariate matrix, $\widehat{B}$ and ${\widehat{\Sigma}}^{-1}$ the maximum likelihood estimators for the fixed effect parameters of the covariates and the inverse of the covariance matrix, respectively, and ${g}_i$ the *i* ^*th*^ genotype vector. In particular, 2 different variant weighting systems were considered. The weights suggested by Wu et al. [8] for use in the sequence kernel association test (SKAT), notated as ${w}_i$ for the *i* ^*th*^ variant, are calculated using a Beta distribution and substantially downweight common variants, (${w}_i\sim Beta\left(MAF;{a}_1,{a}_2\right),\;\mathrm{where}\;{a}_1\;\mathrm{and}\;{a}_2\;\mathrm{are}\;\mathrm{prespecified}\;\mathrm{parameters}\Big)$, while the weights suggested by Price et al. [9], notated as ${w}_i^{*}$, have a less-severe penalty by using $\frac{1}{\sqrt{p_i\Big(1-{p}_i\Big)}}$ where ${p}_i$ is the allele frequency in the controls. Following the example of Liu et al. [1], we also raised the score statistics to differing powers, varying the tests’ statistical power in response to a small proportion of causal variants. In general, raising score statistics to higher powers makes the test more robust to the inclusion of noncausal variants. The following 8 tests were considered:

1. $$ {Q}_2=\sum {w}_i^2{U}_i^2 $$
2. $$ {Q}_{2^{*}}=\sum {w^{*}}_i^2{U}_i^2 $$
3. $$ {Q}_1=\left|\sum {w}_i{U}_i\right| $$
4. $$ {Q}_{1^{*}}=\left|\sum {w^{*}}_i{U}_i\right| $$
5. $$ {Q}_4=\sum {w}_i^4{U}_i^4 $$
6. $$ {Q}_{4^{*}}=\sum {w^{*}}_i^4{U}_i^4 $$
7. $$ {Q}_3=\Big|\sum {w}_i^3{U}_i^3\Big| $$
8. $$ {Q}_{3^{*}}=\Big|\sum {w^{*}}_i^3{U}_i^3\Big| $$

We note that *Q*_*2*_ is asymptotically equivalent to famSKAT [2], the family-based version of SKAT [8], and *Q*_*1*_ is related to standard burden tests.

## Results

### Type I error

Across all 30 genes, for all tests, the type I error rate was generally controlled. The average type I error rate across the 30 genes fell within a range of 0.048 to 0.09 for the 9 different methods, which was within expected limits (99% confidence interval [CI]: 0.4%, 9.6%) (Table 1).

**Table 1:** Type I error rate for 10 genes selected to be representative of all 30 genes explored

Gene	${Q}_2$	${Q}_{2^{*}}$	${Q}_1$	${Q}_{1^{*}}$	${Q}_4$	${Q}_{4^{*}}$	${Q}_3$	${Q}_{3^{*}}$	Combined
High power									
*ARF4*	0.055	0.05	0.015	0.03	0.065	0.04	0.04	0.025	0.03
*DNASE1L3*	0.055	0.085	0.07	0.07	0.08	0.075	0.075	0.07	0.055
*MAP4*	0.06	0.05	0.055	0.09	0.085	0.09	0.055	0.06	0.07
*SCAP*	0.09	0.135	0.025	0.055	0.08	0.18	0.055	0.075	0.055
Low power									
*CXCR6*	0.07	0.045	0.115	0.065	0.075	0.065	0.065	0.045	0.04
*PAK2*	0.04	0.095	0.045	0.055	0.04	0.08	0.035	0.055	0.035
*PTPLB*	0.005	0.025	0.055	0.015	0.01	0.045	0.01	0.035	0.03
*RAD18*	0.045	0.045	0.025	0.06	0.045	0.035	0.04	0.04	0.03
Other representative genes									
*PDCD6IP*	0.05	0.035	0.055	0.045	0.055	0.055	0.05	0.045	0.035
*ZBTB38*	0.065	0.035	0.045	0.02	0.06	0.055	0.055	0.04	0.03
Mean Across All 30 Genes (SD)	0.067 (0.03)	0.074 (0.05)	0.048 (0.02)	0.059 (0.05)	0.07 (0.03)	0.084 (0.05)	0.058 (0.03)	0.065 (0.05)	0.058 (0.04)

Type I error rates are reported for all 9 tests across 10 different genes: the 4 genes with highest overall power, the 4 genes with lowest overall power, and 2 genes deemed representative of the remaining genes explored. The mean type I error rate for each test statistic across all 30 genes is reported at the bottom of the table. Averaged across all 30 genes, the type I error rate was nominally conserved at a 0.05 level

### Power

**Fig. 1:**
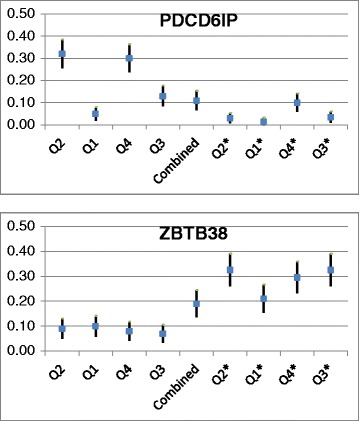
Empirical power of all 9 family-based rare-variant tests of association for 2 genes of interest, *PDCD6IP* and *ZBTB38*. When performing family-based rare-variant tests of association on gene *PDCD61P*, ${w}_i$-weighted tests (Q2, Q1, Q3, Q4) saw higher empirical power than ${w_i}^{*}$-weighted tests (Q2*, Q1*, Q4*, Q3*); for gene *ZBTB38*, this trend was reversed. The combined test (*black*) maintained consistent power between the 2 genes, and was less susceptible to the change in genetic architecture

For nearly all genes analyzed, the empirical power was low, and typically well under 0.35 (Table 2). This was particularly true for those tests with ${Q}_1$ or ${Q}_{1^{*}}$, which had substantially lower power; in the case of ${Q}_1$ this is likely because the ${w}_i$ weights are designed to be squared, whereas in the case of ${Q}_{1^{*}}$ it is likely because common variants were not excluded, and the ${w}_i^{*}$ weights do not sufficiently downweight common variants relative to rare variants. However, despite the presence of these 2 underpowered tests in the combined *p* value procedure, the combined test had power in the same range as more optimal tests. We further note that, depending on the characteristics of the gene being examined, tests featuring either the ${w}_i^{*}$ weights or the ${w}_i$ weights were often dramatically more powerful than the other. This can be attributed to the fact that ${w_i}^{*}$ less dramatically downweights common variants relative to rare variants than does ${w}_i$. This phenomenon is particularly evident in 2 genes: *PDCD6IP*, which contains a number of highly causal rare variants, and *ZBTB38*, which contains several causal common variants (Fig. 1). In each case, however, the combined test strikes a middle ground: not quite as strong as the better-chosen weights, but superior to the poorer weights (Fig. 1).

**Table 2:** Power for 10 genes selected to be representative of all 30 genes explored

Gene	${Q}_2$	${Q}_{2^{*}}$	${Q}_1$	${Q}_{1^{*}}$	${Q}_4$	${Q}_{4^{*}}$	${Q}_3$	${Q}_{3^{*}}$	Combined
High power									
*ARF4*	0.425	0.315	0.07	0.075	0.885	0.585	0.785	0.435	0.58
*DNASE1L3*	0.525	0.315	0.13	0.055	0.53	0.365	0.455	0.365	0.325
*MAP4*	0.995	0.995	0.225	0.99	0.995	0.995	0.995	0.995	0.995
*SCAP*	0.995	0.82	0.54	0.17	0.99	0.98	0.985	0.78	0.945
Low power									
*CXCR6*	0.025	0.015	0.005	0.01	0.025	0.03	0.025	0.035	0.025
*PAK2*	0.04	0.025	0.04	0.005	0.035	0.045	0.045	0.015	0.025
*PTPLB*	0.02	0.0	0.025	0.035	0.01	0.005	0.015	0.025	0.01
*RAD18*	0.025	0.005	0.05	0.04	0.005	0.005	0.02	0.015	0.01
Other representative genes									
*PDCD6IP*	0.32	0.03	0.05	0.015	0.30	0.10	0.13	0.035	0.11
*ZBTB38*	0.09	0.325	0.10	0.21	0.08	0.295	0.07	0.325	0.19
Mean Across All 30 Genes (SD)	0.18 (0.25)	0.18 (0.23)	0.08 (0.11)	0.11 (0.18)	0.22 (0.30)	0.20 (0.25)	0.17 (0.27)	0.17 (0.23)	0.17 (0.25)

Statistical power is reported for all 9 tests across 10 different genes: the 4 genes with highest overall power, the 4 genes with lowest overall power, and 2 genes deemed representative of the remaining genes explored. The mean power for each test statistic across all 30 genes is reported at the bottom of the table

### Correlation between tests

Pairwise correlation between *p* values of different tests was generally high with an average pairwise correlation of 0.78 (SD = 0.16; min = 0.42; max = 0.98; detailed results not shown).

## Discussion

Few current methods allow for a flexible combination of family-based rare-variant tests of association. In response, we propose an adaptable framework for combining any number and type of gene-based rare-variant tests on pedigree data that condition on genotype data. Unlike current existing combination methods, our proposed method does not limit the researcher to combining just 1 family-based burden test with 1 family-based variance-component test, but instead allows for the combination of an arsenal of varied and specialized tests. Our proposed method also offers a distinct advantage over simply running all possible individual tests and choosing the most significant result; our combination approach appropriately controls for multiple testing and for the correlation between tests. When applied to the GAW19 data, the combination method appeared to sufficiently maintain type I error and demonstrated generally robust statistical power, even when applied to a wide variety of genetic architectures, or when low-powered tests were included.

However, our current approach does have several limitations. In the above proof-of-concept application, the combined test incorporates 8 highly correlated individual tests of association; the additional noise from this correlation diminishes the power of the combined test to detect any meaningful genotype–phenotype associations. Further research is needed to explore what combination of tests minimizes such correlation while also maximizing statistical power. Further work is also necessary to evaluate the benefits and drawbacks of different choices of combined test statistics (eg, alternatives to using the minimum *p* value).The permutation component of our combination approach also poses computational challenges. The process of calculating *p* values when asymptotic distributions have not been derived is time intensive. The tests evaluated here were applied to only 30 genes in a single simulated data set. We note that if combining tests with known asymptotic distributions, it is straightforward to utilize asymptotically obtained *p* values from individual tests when creating ${\boldsymbol{p}}^{(j)}$. Further testing and evaluation on other data sets with different characteristics is necessary. A single family-based rare-variant test of association, if properly chosen and adapted to match the genetic architecture, will always be more powerful than a combined approach. That being said, researchers may not definitively know what the underlying genetic architecture is, and there is always a tradeoff between power and robustness. Given this, combined tests on family-based rare-variant data show promise for ensuring the robustness of statistical power to different genetic architectures with minimal power loss. Finally, the permutation approach proposed here relies on the assumption that the regression model is true under the genetic null hypothesis, which will rarely be true in practice. However, both Chen et al. [2] and our results suggest that the type I error rate is controlled in practice. Further research is necessary to explore whether samples and conditions exists where the type I error rate will increase.

## Conclusions

Our analysis acts as a proof-of-concept for combined tests on family-based rare-variant data. In particular, our analysis suggests that the type I error rate is controlled by the proposed method, while power may be more robust to differing genetic architectures than individual, gene-based rare-variant tests. Further work is needed to explore the trade-off between combining many tests versus combining a smaller set of diverse tests in other data sets/genes to ensure transferability of these findings. Additionally, we need to explore alternatives to permutation strategies to compute statistical significance resulting from the inherent computational limitations of this approach.
